# Structure Determination of Au on Pt(111) Surface: LEED, STM and DFT Study

**DOI:** 10.3390/ma8062935

**Published:** 2015-05-27

**Authors:** Katarzyna Krupski, Marco Moors, Paweł Jóźwik, Tomasz Kobiela, Aleksander Krupski

**Affiliations:** 1 Department of Physics, University of Warwick, Coventry CV4 7AL, UK; E-Mail: k.j.krupski@warwick.ac.uk; 2 Peter Grünberg Institut, Forschungszentrum Jülich, Wilhelm-Johnen-Str., 52425 Jülich, Germany; E-Mail: m.moors@fz-juelich.de; 3 Department of Advanced Materials and Technologies, Faculty of Advanced Technologies and Chemistry, Military University of Technology, Kaliskiego 2 Str., 00-908 Warszawa, Poland; E-Mail: pjozwik@wat.edu.pl; 4 Faculty of Chemistry, Warsaw University of Technology, ul. Noakowskiego 3, 00-664 Warsaw, Poland; E-Mail: kobiela@ch.pw.edu.pl; 5 Faculty of Science, SEES, University of Portsmouth, Portsmouth PO1 3QL, UK

**Keywords:** density functional theory calculations, scanning tunneling microscopy, low-energy electron diffraction, surface structure, metallic surfaces, gold, platinum, metal-metal interfaces, low index single crystal surface

## Abstract

Low-energy electron diffraction (LEED), scanning tunneling microscopy (STM) and density functional theory (DFT) calculations have been used to investigate the atomic and electronic structure of gold deposited (between 0.8 and 1.0 monolayer) on the Pt(111) face in ultrahigh vacuum at room temperature. The analysis of LEED and STM measurements indicates two-dimensional growth of the first Au monolayer. Change of the measured surface lattice constant equal to 2.80 Å after Au adsorption was not observed. Based on DFT, the distance between the nearest atoms in the case of bare Pt(111) and Au/Pt(111) surface is equal to 2.83 Å, which gives 1% difference in comparison with STM values. The first and second interlayer spacing of the clean Pt(111) surface are expanded by +0.87% and contracted by −0.43%, respectively. The adsorption energy of the Au atom on the Pt(111) surface is dependent on the adsorption position, and there is a preference for a hollow *fcc* site. For the Au/Pt(111) surface, the top interlayer spacing is expanded by +2.16% with respect to the ideal bulk value. Changes in the electronic properties of the Au/Pt(111) system below the Fermi level connected to the interaction of Au atoms with Pt(111) surface are observed.

## 1. Introduction

A large number of studies on epitaxy have been carried out for many years. Ultra-thin epitaxial film systems exhibit a variety of interesting properties due to the strong correlation between the electronic structure of the film and its morphology, strain, and defect structure [1,2,3,4,5,6,7,8,9,10]. Structural studies of *fcc/fcc* systems provide a great deal of information on the connection between the geometrical properties of the adsorbed atomic layers and the atomic arrangements of the substrates. Various fields are concerned with epitaxial growth; these range from basic research on the growth mechanism of thin films to advanced research on the development of devices. Platinum is widely used as a catalyst in the chemical and petrochemical industries [11,12]. For example, in oil refineries, platinum catalysts are employed in processes that involve the reforming of paraffin and the hydrogenation of unsaturated hydrocarbons [11,12,13,14].

The clean Pt(111) surface itself has been the subject of several structural determinations with Low Energy Electron Diffraction (LEED) [15,16,17,18,19,20,21,22,23], medium energy ion scattering (MEIS) [24], high energy ion scattering (HEIS) [25] and surface X-ray diffraction (SXRD) [26,27]. Differently from the other noble metal (111) surfaces, clean Pt(111) normally has an unreconstructed bulk-periodic surface [23,28,29,30]. Adams *et al.* [18] found the first layer spacing to be possible to expand by 0.04 ± 0.10 Å, while Hayek *et al.* [21] found an unrelaxed surface with 0.05 Å. Materer *et al.* [23] found the first and second interlayer spacing expanded by 0.04 ± 0.10 Å and 0.005 ± 0.03 Å, respectively. MEIS and HEIS experiments [24,25] support the LEED results. Namely, the ion scattering data indicate that the Pt(111) structure deviates from the bulk geometry by a possible small outward expansion of the top interlayer spacing of 0.03 ± 0.02 Å [24] or 0.03 ± 0.02 Å [25]. No deviation from the bulk position was found in the direction parallel to the surface, with a small accuracy of about 0.01 ± 0.02 Å. The surface geometry of clean Pt(111) has been the subject of surface X-ray diffraction investigations [26]. These investigations gave an outward relaxation of the topmost layer of 0.045 ± 0.005 Å (+2.0%) with respect to the ideal bulk termination.

Properties of ultrathin gold layers deposited on the Pt(111) face were investigated in a number of works [31,32,33,34,35,36,37,38,39,40,41,42]. Studies on single crystalline Au-Pt(111) model surfaces, for instance, have provided detailed information on the catalytic properties of these surfaces [31,32,33]. Davies *et al.* [31] studied the growth and chemisorptive properties of gold and silver monolayers on platinum (111) and (553) single crystal surfaces using Auger electron spectroscopy (AES), LEED, and temperature-programmed desorption (TPD). The AES results suggested that the growth of Au proceeds via a Stranski-Krastanov mechanism at room temperature, and that at temperatures above 800 K gold dissolves into the Pt crystal bulk. No extra LEED order spots or spot streaking was observed. In contrast, Shatler *et al.* [32] with the use of AES, LEED, and TPD found that deposition of gold on Pt(111) near T = 300 K indicates a layer-by-layer (Frank-van der Merwe) growth mechanism up to three gold monolayers. The analysis of AES measurements showed that two-dimensional islands growth below one monolayer took place. Furthermore, with increasing coverage, the gold islands grew until the monolayer is completed, before the second layer begins to form. In additional studies by Sachtler *et al.* [33], the activity for conversion of n-hexane as a function of Au surface concentration on Pt(111) was monitored. The Au-covered crystal was then annealed at elevated temperatures to allow Au intermixing with the Pt substrate. The formed Au-Pt(111) surface alloy showed a much higher activity for n-hexane isomerization than pure Pt. Moreover, it has been reported that Au in a dispersed state exhibits a high activity for some reactions at low temperatures (e.g., CO oxidation) [35] and that this feature depends on the preparation conditions, size and shape of the Au nanostructures [36]. Adsorption experiments with CO as a titration agent showed a significantly lower affinity of the Au-Pt surface alloy in comparison to the clean Pt surface [37]. Salmeron *et al.* [38] used photoelectron spectroscopy techniques (UPS (ultraviolet) and XPS (X-ray)), LEED and AES to study the electronic structure of Au and Ag overlayers deposited on Pt(111), Pt(100), and Pt(997). Between 0 and 1 monolayer, the valence bands of Au and Ag show changes in the form of shifts of the most tightly bound peaks and the appearance of the new structures around a coverage (θ_Au_) of one monolayer. The Au 5*d*_3/2_ peak shifts 0.6 eV towards higher binding energies when coverage varies from 0.1 to 1 monolayer and 0.5 eV more when coverage varies from one to six monolayers. These shifts are explained as due to the changing contributions of the Au atoms in island edges for surface (θ_Au_ < 1) monolayer and bulk (θ_Au_ > 1) coordination positions. Using AES, they found that gold on Pt(111) grows layer-by-layer. Below θ_Au_ < 1, no extra LEED spots were observed. In addition, the work function decreased upon gold deposition from its initial value of 6.08 ± 0.15 eV for clean Pt(111) down to 5.8 ± 0.15 eV. That value was reached at the monolayer and remained constant thereafter up to five monolayers and is clearly larger than the 5.31 eV value reported by Potter *et al.* [43] for bulk Au(111). The work function for the Pt(111) surface compares only fairly with that reported by Ertl *et al.* [44] of 6.40 eV. Its smaller value might reflect a less perfect surface with larger number of residual steps. It should be pointed out here that Pt(111) surface presents the highest work function value among other metals surfaces. Vogt *et al.* [39] studied Au/Pt(111) system by spin-, angle- and energy-resolved photoemission with normal incident circularly polarized synchrotron radiation of BESSY and normal photoelectron emission for different Au coverages. The prepared layers were characterized by AES and LEED and turned out to grow up two-dimensionally and epitaxially. LEED spots did not show any changes in geometry during the evaporation time up to the coverage of a thick Au layer [39]. Later, the electrodeposition of Au on Pt(111) from electrolytes containing µM concentrations of $AuCl_{4}^{-}$ was investigated by *in situ* electrochemical scanning tunneling microscopy (EC-STM) by Sibert *et al.* [41,42]. Under conditions of high Au surface mobility, multilayer growth proceeds via a typical Stranski-Krastanov growth mode, with layer-by-layer growth of a pseudomorphic Au film up to two monolayers and three-dimensional growth of structurally relaxed islands at higher coverage, indicating thermodynamic control under these conditions.

In the present work, in order to study the structural and electronic properties during the initial adsorption process of gold on Pt(111) surface at room temperature, we have performed low-energy electron diffraction, scanning tunneling microscopy measurements in ultrahigh vacuum and density functional theory calculations with the use of CASTEP code.

## 2. Experimental Details

The measurements were carried out in a stainless steel ultra-high vacuum chamber with a base pressure of 2.0 × 10^−8^ Pa. The chamber was equipped with a reverse-view LEED optics, which was used for low-energy electron diffraction measurements, and also with a variable-temperature scanning tunneling microscopy stage. The Pt(111) single crystal was supplied by MaTeck [45]. The surface of the Pt(111) single crystal was cleaned by repeated cycles of sputtering with 3 keV Argon ions at T = 300 K and annealing at T = 1100 K. After annealing at 1100 K, the residual carbon was removed in 7.0 × 10^−4^ Pa of oxygen, followed by desorption of any remaining oxygen at 1200 K. This procedure was repeated until the LEED pattern of a clean Pt(111) surface with sharp spots and low background was obtained. The deposition of Au (99.999%) on the Pt(111) sample was achieved by vaporization from a Knudsen cell and the coverage of gold was determined via STM. Film coverages are described in monolayers (ML), where a 1 ML Pt(111) film corresponds to an atomic packing density of 1.503 × 10^15^ atoms/cm^2^ obtained from a bulk lattice constant a_Pt_ = 3.9239 Å [46] (for comparison the atomic packing density of Au(111) equals 1.387 × 10^15^ atoms/cm^2^ for a_Au_ = 4.0785 Å [46]). This cell had been constructed from an Al_2_O_3_ crucible from Friatec [47] with a diameter of 5 mm. It was filled with a 0.5 mm thick Au wire from Goodfellow [48] and closed by a two-hole ceramic. The Knudsen cell was heated by a tungsten wire from Goodfellow (diameter 0.3 mm) wound around the crucible and thermally shielded by a water-cooled jacket. In order to control the deposition time, a rotatable shutter was placed in front of the cell opening. The working pressure during Au deposition was below 1.0 × 10^−7^ Pa. All STM measurements were performed with the use of electrochemically etched W (99.99%) tips (diameter 0.5 mm, length 3.5 mm). For the potassium hydroxide electrolyte, a 4 V_p−p_ square wave voltage (*f* = 100 Hz) was applied to the tip. In the electrochemical cell, a tungsten wire is used as the working electrode (anode) and a Pt (99.999%) loop (diameter 10 mm) is used as the counter electrode (cathode). A 3 M KOH solution from Sigma Aldrich [49] is used as the electrolyte. The following reactions take place:

Cathode Pt (Reduction Reaction):

(1) $$6e^{-}+6{\text{K}}^{+}+6{\text{H}}_{\text{2}}\text{O}+{\text{Pt}}_{\text{CATALYSTS}}\to 6\text{KOH}+3{\text{H}}_{\text{2}}+{\text{Pt}}_{\text{CATALYSTS}}$$

Anode W (Oxidation Reaction):

(2) $$\text{W}\to {\text{W}}^{6+}+6e^{-}$$

(3) $${\text{W}}^{6+}+6\text{KOH}\to {\text{W(OH)}}_{\text{6}}+6{\text{K}}^{+}$$

Total Reaction:

(4) $$\text{W}+6{\text{H}}_{\text{2}}\text{O}+6\text{KOH}+{\text{Pt}}_{\text{CATALYSTS}}\to {\text{W(OH)}}_{6}+3{\text{H}}_{\text{2}}+6\text{KOH}+{\text{Pt}}_{\text{CATALYSTS}}$$

**Figure 1:**
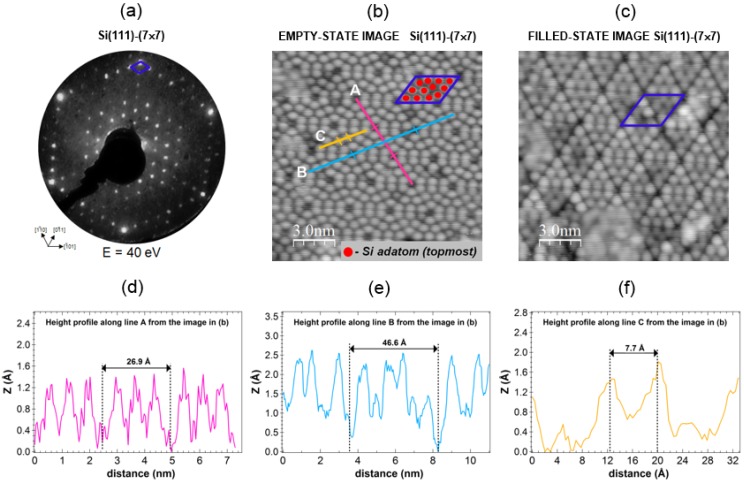
Si(111)-(7 × 7) surface at T = 300 K: (**a**) LEED patterns recorded at normal electron incidence for E = 40 eV; (**b**) STM image of empty states (150 Å × 150 Å, I_T_ = 0.5 nA, U_bias_ = +1.6 V); and (**c**) STM image of filled states (150 Å × 150 Å, I_T_ = 0.5 nA, U_bias_ = −1.6 V). Au deposited on Pt(111) at T = 300 K at a coverage θ_Au_ ≤ 1.0 ML: (a) θ_Au_ ≈ 0.8 ML (5000 Å × 5000 Å, I_T_ = 4.0 nA, U_bias_ = 1.0 V); (**d**–**f**) line scans along the lines A, B, and C from the image in (b). The unit cell is indicated by the blue diamond (diagonals: a_1_ = 46.6 Å, a_2_ = 26.9 Å). Si adatoms (12 per surface unit cell) cell are marked as red dots.

All presented STM images were recorded in constant current mode and processed by the WSXM image-processing software [50]. Before starting experimental investigations of the Pt(111) and Au-Pt(111) surfaces, the experimental system was calibrated with the use of well know Si(111)-(7 × 7) reconstructed surface [51,52,53,54] (Figure 1). Si(111)-(7 × 7) surface was prepared by twice direct current flashing (I = 4.0 A) an *p*-type Si(111) substrate (size: 1 × 10 × 0.5 mm, resistivity ρ ≈ 1–10 Ω cm) at 1220 K, after degassing at 970 K for two hours by joule heating with a current of 1 A. Atomically resolved STM images of the empty and filed states of Si(111)-(7 × 7) are presented in Figure 1b,c, respectively. The measured surface unit cell is characterized by two diagonals of the diamond (a_1_ = 46.6 Å and a_2_ = 26.9 Å). Silicon adatoms (12 per unit cell) are marked in red in Figure 1b, and they occur as bright “dots” in empty-state STM image. Visible in STM images deep holes (depth ~ 2 Å) are called corner hols.

## 3. Calculation Details

All calculations were performed based on the pseudo-potential plane-wave within the density functional theory [55,56], using the Cambridge serial total energy package (CASTEP) [57]. The effects of exchange correlation interaction are treated with the generalized gradient approximation (GGA) of Perdew-Burke-Ernzerhof (PBE) [58,59]. The ultra-soft pseudo-potentials [60] describe this electron-ion interaction system to high accuracy with a plane wave energy cutoff of 600 eV. The energy calculations in the first irreducible Brillouin-zone were conducted by using the (4 × 4 × 1) k-point grid of the Monkhorst-Pack scheme [61]. Spin polarization of platinum was included in the calculations to correctly account for its magnetic properties. All atomic positions have been relaxed according to the total energy and force using the BFGS scheme [62] based on the cell optimization criterion RMS force of 0.03 eV/Å, stress of 0.05 GPa, and displacement of 0.001 Å. The calculation of total energy and electronic structure is followed by cell optimization with SCF tolerance of 1 × 10^−6^ eV/atom. The Pt(111) surface was modeled using a slab containing 7 (=15.84 Å) and 10 (=22.63 Å) layers of Pt atoms with a vacuum gap in the [111] direction equal to 20.57 Å and 30.37 Å, respectively. Full slab relaxation was performed in both cases.

## 4. Results and Discussion

### 4.1. LEED and STM

**Figure 2:**
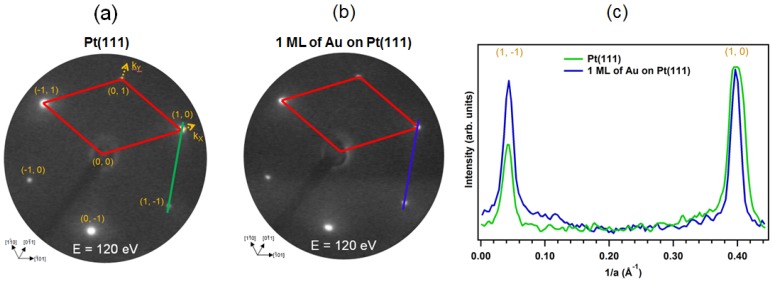
LEED patterns observed during the growth of Au on the Pt(111) surface recorded at normal electron incidence for E = 120 eV, and T = 300 K: (**a**) clean Pt(111) for E = 120 eV. *k_X_* and *k_Y_* denote axes in the reciprocal lattice; (**b**) 1 ML of Au on Pt(111); and (**c**) line profile along the lines from the image in (**a**,**b**) demonstrating that the position of LEED spots remain unchanged after gold deposition. The unit cell is outlined.

Gold atoms on the Pt(111) face form an ordered structure after evaporation onto the crystal face. Typical LEED pattern observed before and after deposition of gold on the Pt(111) face in normal electron incidence are shown in Figure 2. In this figure, the unit cell of the platinum lattice is indicated. The lattice constant of the platinum surface unit cell is 2.775 Å (primitive *fcc* (111) unit cell) [63]. The patterns are shown to demonstrate the quality of the structural order at the surface. It should be pointed out that the positions of the LEED spots associated with the Pt(111) substrate remains unchanged during the gold deposition at 300 K (Figure 2c), as was previously reported by Sachtler and Samorjai [32] and Vogt *et al.* [40]. Thus, the lattice constant of the first substrate layer remains constant, too, and suggests a two-dimensional growth of the first gold layer.

**Figure 3:**
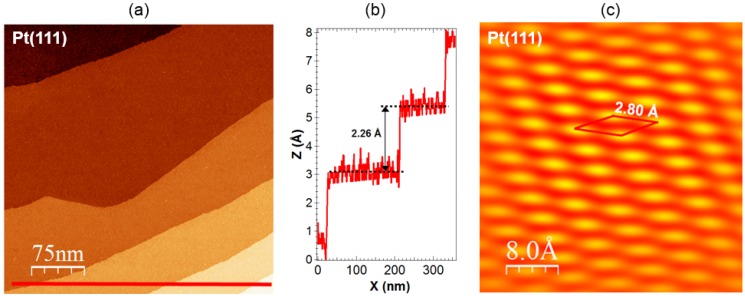
STM images of the clean Pt(111) surface: (**a**) T = 25 K (3734 Å × 3734 Å, I_T_ = 141 pA, U_bias_ = +50 mV); (**b**) line-scan corresponding to line drawn in (**a**); and (**c**) T = 300 K (40 Å × 40 Å, I_T_ = 49 pA, U_bias_ = +48 mV). The unit cell is outlined. STM image evidences a hexagonal lattice arrangement of Pt atoms with measured nearest neighbor distance of 2.80 Å.

**Figure 4:**
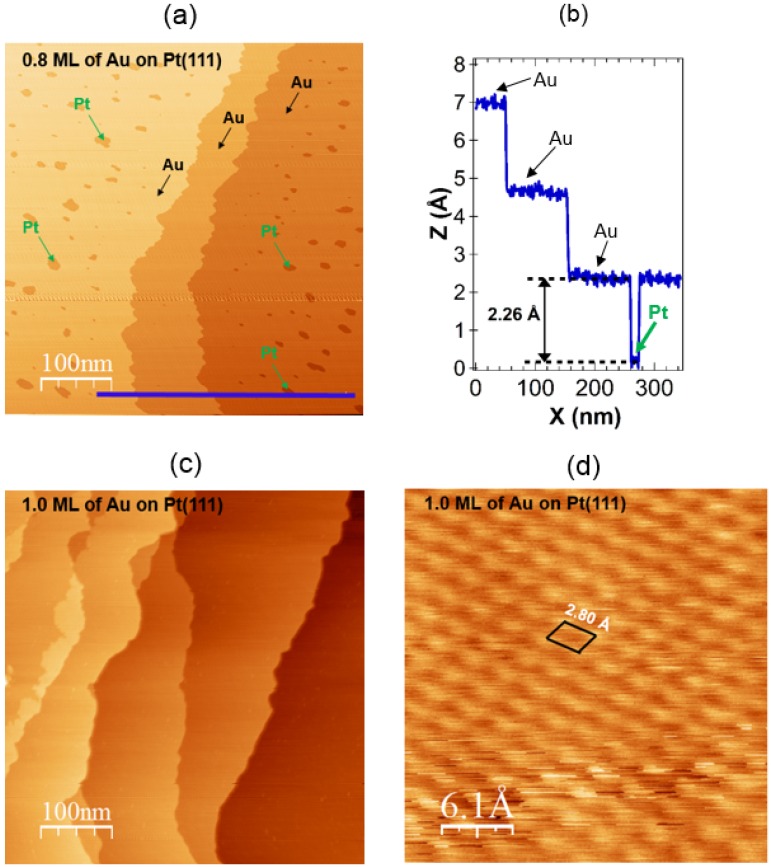
STM images of Au deposited on Pt(111) at T = 300 K at a coverage θ_Au_ ≤ 1.0 ML: (**a**) θ_Au_ ≈ 0.8 ML (5000 Å × 5000 Å, I_T_ = 4.0 nA, U_bias_ = +1.0 V); (**b**) line scan along the line from the image in (a) demonstrating that the height of the gold layer corresponds to the height of a single Pt step height; (**c**) θ_Au_ ≈ 1.0 ML (5000 Å × 5000 Å, I_T_ = 2.0 nA, U_bias_ = +1.0 V); and (**d**) (30 Å × 30 Å, I_T_ = 4.65 nA, U_bias_ = +159 mV). The unit cell is outlined. STM image evidences a hexagonal lattice arrangement of Au atoms with measured nearest neighbor distance of 2.80 Å.

The results of our STM measurements on the clean Pt(111) surface are presented in Figure 3. Figure 3a displays an STM image, taken on a low-index Pt(111) substrate with terraces between 100 and 300 nm width separated by monoatomic steps. The height of the steps on the Pt(111) surface was measured by STM to be 2.26 ± 0.3 Å (Figure 3b). However, one need to remember that the observed by STM step height includes geometric and electronic factors. Figure 3c presents atomic resolution of the Pt(111) face. The obtained topography shows a hexagonal lattice arrangement of Pt atoms with the nearest neighbor distance of 2.80 Å. This value describes the dimension of the surface unit cell and is 0.90% higher compared to the literature value (=2.775 Å) [63]. Figure 3c demonstrates that the surface structure seen in the obtained STM image has a clear long-range character. Figure 4 shows STM images of the Pt(111) surface with varying Au coverage in order to illustrate the morphology of the Au layers deposited on Pt at room temperature. Figure 4a shows a typical STM image corresponding to a submonolayer coverage of θ_Au_ ≈ 0.8 ML. An analysis of the STM measurements indicates that for coverage less than 1 ML, two-dimensional growth of gold layer is observed. This is in agreement with photoelectron spectroscopy study [38], our present and previous AES/LEED measurements [32,39]. The darker features in Figure 4a represent still visible platinum substrate as predicted in the previous studies [32]. Similar to the results observed by us, two-dimensional gold monolayer was obtained by electrodeposition of Au on Pt(111) from electrolytes containing µM concentrations of of $AuCl_{4}^{-}$ [42]. The line scan in Figure 4b shows that the height of the first gold layer corresponds to the height of a single Pt step height equal to 2.26 Å. As the Au coverage is close to 1 ML, Au wets the Pt(111) surface completely, as can be seen in Figure 4c. This is not easy to confirm with STM, whether the surface is wetted or not. However, the reason for the perfect wetting is because of the high-specific surface free energy of the Pt(111) surface (2.299 J/m^2^ < γ_Pt(111)_ < 2.489 J/m^2^) [64,65,66,67,68] as compared with that of the Au(111) surface (1.283 J/m^2^ < γ_Au(111)_ < 1.506 J/m^2^) [64,65,66,67,68]. Since the total specific surface free energy should be minimized, a covered Pt(111) surface is favored [69]. Closer view of the STM image topography in Figure 4d reveals the presence of well-ordered gold structures. STM images indicate a long-range order in the surface system. The obtained topography shows a hexagonal lattice arrangement of Au atoms with a nearest neighbor distance of 2.80 Å, which is exactly the same value as mentioned above in the case of Pt atoms. The same value of the surface unit cell after adsorption of gold could suggest that the gold atoms are adsorbed in sites (hollow *fcc* or *hcp*), which are a direct continuation of the Pt lattice *ABCABCA*. This is in good agreement with the supposition from the spin-resolved photoemission studies of Au-Pt(111) system [40], where the best fit of experimental results and theoretical model was achieved on that basis.

### 4.2. DFT

#### 4.2.1. Structure of Clean Pt(111)

**Figure 5:**
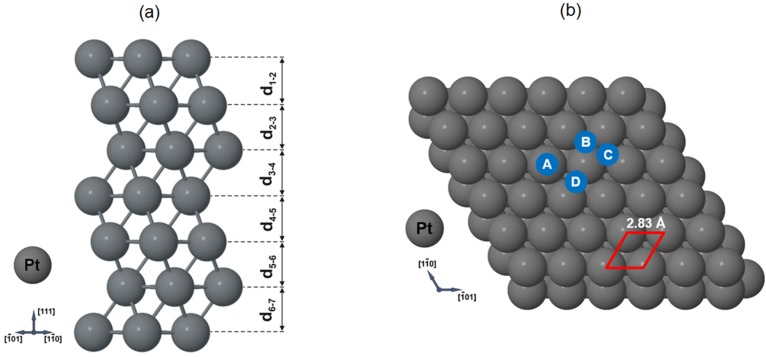
(**a**) Side view of the relaxed Pt(111) surface for seven layers. Values of denoted characteristic inter plane distances are given in Table 1. (**b**) Considered positions of Au adsorption on the Pt(111) surface: A—on top; B—hollow *fcc*; C—hollow *hcp*; and D—bridge. The unit cell is outlined. The nearest neighbor Pt-Pt distance of 2.83 Å is obtained from our theoretical calculations.

In the theoretical part of our work, we have calculated multilayer relaxations of the Pt(111) system using the slab with 7 and 10 atomic layers. Figure 5a shows the schematic view of relaxed slab structure for the seven platinum layers. The platinum low-index surface was modeled by repeated slabs with a (1 × 1) surface unit cell with four atoms in each layer. The calculated atomic layer distances for seven and ten planes are shown in Table 1. $d_{i-j}^{X-Y}$ defines the distance along the surface normal direction between the *X* atom at the *i* atomic layer and the *Y* atom at the *j* atomic layer. Surface relaxation $\Delta d_{i-j}^{X-Y}$ is characterized as the percent of change of the spacing between layers *i* and *j versus* the bulk layer spacing (d^0^). Bulk value (d_0_) is taken from our GGA calculations and describes average distance between atomic planes of seven (=2.30 Å) and ten (=2.29 Å) platinum layers, respectively. Further calculations of gold adsorption on Pt(111) surface has been performed on seven platinum layers.

Our calculations for the clean Pt(111) show very good agreement with the above-presented STM results and with the other experimental and theoretical literature studies [15,16,18,20,21,22,23,26,30,70,71] presented in Table 1. Obtained lateral geometrical properties of Pt(111) surface and distances between the nearest Pt atoms in the structure (=2.83 Å) are very close to STM measurements (=2.80 Å), with the difference about 1%. The first and second interlayer spacings of the clean Pt(111) surface were determined to be 2.32 Å and 2.29 Å, respectively, in case of calculated slab with seven atomic layers. This corresponds to a +0.87% expansion and −0.43% contraction of the first and second metal layer spacings of the ideally terminated Pt(111) clean surface (=2.30 Å), respectively. These values and the value obtained in our calculations of lattice constant of the bulk Pt (=3.99 Å) are in excellent agreement with the previous GGA calculations [70] and surface X-ray diffraction results [26] (Table 1). However, comparison of our calculations to the quantitative low-energy electron diffraction value of the first interlayer spacing shows that our theoretical value (=2.32 Å) is slightly larger (+2%) then the average value of 2.27 Å observed experimentally [15,16,18,20,21,22,23].

**Table 1:** Distances ($d_{i-j}^{X-Y}$) between the atomic planes of the relaxed Pt(111) system, and their percentage changes ($\Delta d_{i-j}^{X-Y}$) with respect to the bulk value (d_0_), calculated for the slab with 7 and 10 atomic layers and compared with experimental [15,16,18,20,21,22,23,26,72] and theoretical [30,70,71] literature data. Notation of inter-plane distances are the same as in Figure 1.$\;d_{i-j}^{X-Y}$ denotes the interlayer spacing between layers *i* and *j* for the *X* and *Y* atoms type. d_0_—average distance between atomic planes of seven and ten layers, respectively. a_0_—lattice constant of Pt. GGA—generalized gradient approximation, LDA—local density approximation, LEED—low energy electron diffraction, SXRD—surface X-ray diffraction.

Present Work GGA *(CASTEP)*					Previous DFT study		LEED	SXRD ^g^	
$d_{i-j}^{x-y}$(Å)	7 layers	$\Delta d_{i-j}^{x-y}$(%)	10 layers	$\Delta d_{i-j}^{x-y}$(%)	$d_{i-j}^{x-y}$(Å)	$\Delta d_{i-j}^{x-y}$(%)	$d_{i-j}^{x-y}$(Å)	$d_{i-j}^{x-y}$(Å)	$\Delta d_{i-j}^{x-y}$(%)
$d_{1-2}^{Pt-Pt}$	2.32	+0.87	2.33	+1.75	2.766 *^h^*(LDA)		2.26 *^a^*	2.31 ± 0.05	+2.0
						+0.44 *^h^*(LDA)	2.29 ± 0.1 *^b^*		
						+0.85 *^i^*(LDA)	2.2713 *^c^*		
						+1.14 *^j^*(LDA)	2.26 ± 0.05 *^d^*		
						+0.85 *^j^*(LDA)	2.2655 ± 0.025 *^e^*		
							2.29 ± 0.001 *^f^*		
$d_{2-3}^{Pt-Pt}$	2.29	−0.43	2.29	-	2.746 *^h^*(LDA)	−0.31 *^h^*(LDA)	2.26 *^a^*		
						−0.56 *^i^*(GGA)	2.2405 ± 0.025 *^e^*		
						−0.29 *^j^*(LDA)	2.27 ± 0.003 *^f^*		
						−0.22 *^j^*(LDA)			
$d_{3-4}^{Pt-Pt}$	2.30	-	2.29	-		−0.15 *^i^*(GGA)	2.26 *^a^* 2.2655 ± 0.05 *^e^*		
						−0.21 *^j^*(LDA)			
						−0.17 *^j^*(LDA)			
$d_{4-5}^{Pt-Pt}$	2.30	-	2.30	+0.43			2.26 *^a^*		
$d_{5-6}^{Pt-Pt}$	2.29	−0.43	2.29	-					
$d_{6-7}^{Pt-Pt}$	2.30	-	2.30	+0.43					
$d_{7-8}^{Pt-Pt}$			2.29	-					
$d_{8-9}^{Pt-Pt}$			2.29	-					
$d_{9-10}^{Pt-Pt}$			2.30	+0.43					
$d_{0}\left(Å\right)$	2.30		2.29		2.75 *^h^*(LDA)		2.26 *^c^*		
							2.2655 *^e^*	2.26	
							2.265 *^f^*		
$a_{0}\left(Å\right)$	3.99		3.99		3.99 *^i^*(GGA)	3.92 *^k^*(EXP)	3.92 *^a^* 3.9231 *^d^*		
					3.97 *^j^*(LDA)				
					3.89 *^j^*(LDA)				

*^a^* Ref. [15,16]; *^b^* Ref. [18]; *^c^* Ref. [20]; *^d^* Ref. [21]; *^e^* Ref. [22]; *^f^* Ref. [23]; *^g^* Ref. [26]; *^h^* Ref. [30]; *^i^* Ref. [70]; *^j^* Ref. [71]; *^k^* Ref. [72].

#### 4.2.2. Structure of the Au/Pt(111) System

Figure 5b shows the four possible gold adsorption sites on the Pt(111) surface with one on-top site (labeled as A), two hollow sites: hollow *fcc* (labeled as B), hollow *hcp* (labeled as C), and one bridge site (labeled as D), In our calculations, we define one monolayer of adsorbed Au atoms corresponding to the same atoms as the atomic sites in the surface layer. One Au atom adsorbing on the Pt(111) surface corresponds to an adsorption coverage of 0.25 ML. The minimum adsorption energy (E_ads_) was calculated by means of the following total energy difference:

(5) $$E_{ads}=\;E_{T}\left(\frac{Au}{Pt\left(111\right)}\right)-E_{T}\left(Au\right)-E_{T}\left(Pt\left(111\right)\right)$$

where *E_T_* is the total energy of the system and $\frac{Au}{Pt\left(111\right)}$, Au, and Pt(111) refer to the atom-on-metal system, the free Au atom, and the bare Pt surface, respectively.

**Figure 6:**
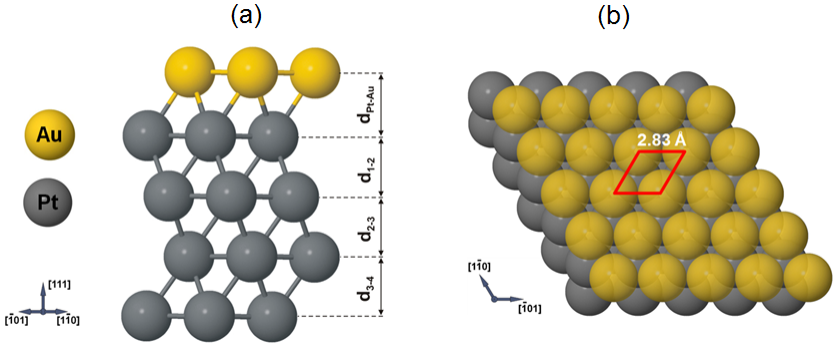
(**a**) Side view of the calculated most stable hollow *fcc* position of a relaxed Au atom on the Pt(111) surface; (**b**) top view of the calculated Au/Pt(111) surface. The unit cell is outlined. The nearest neighbor Au-Au distance of 2.83 Å is obtained from our theoretical calculations.

Table 2 displays the predicted adsorption energies of Au on the Pt(111) surface and the distance between the Au atom and its nearest (*r_NN_*) and next nearest neighbors (*r_NNN_*). A, B, C and D describe positions of Au atom on the Pt(111) surface before starting calculations. As one can see, only in the case of bridge position D displacement of gold atom towards hollow *fcc* position B is observed, while the other gold adsorption positions described as A, B and C remain unchanged. The comparison of the calculated adsorption energies reveals that the preferred position of the Au on the Pt(111) surface is the hollow *fcc* with the E_ads_ = −0.578 eV. At this favorable position, the nearest to the nearest (NN) and next-nearest (NNN) neighbor distance is equal to 2.58 Å and 3.76 Å, respectively. The adsorption energy of one gold atom in hollow *fcc* site is negative, which indicates in addition that this adsorption position is the most stable. Similar conclusion was obtained in case of a quantitative LEED analysis of the structure of Pt(111) (√3 × √3) R30°-S, where the best agreement between experiment and theory has been found for a model with a sulfur atom in the three-fold hollow *fcc* site [21]. Moreover, our theoretical studies are in agreement with the spin-resolved photoemission predictions where the Au is adsorbed in sites, which are a direct continuation of the Pt lattice [40]. In contrast to very stable hollow *fcc* site, the on-top adsorption position is the most unstable place with E_ads_ = +0.580 eV. Next, taking into account our experimental STM results, we have considered structural model of the Au/Pt(111) surface reproducing in the best way the topography of the obtained STM images. Structural relaxation has shown that such a model is stable. The lateral positions of all gold atoms in the relaxed structure remained the same as in the starting configuration. This model assumes that the gold structure is built up by Au hollow *fcc* and hollow *hcp* atoms (Figure 6). The obtained lateral geometrical properties of this Au/Pt(111) model and distances between the nearest gold atoms in the structure (=2.83 Å) are almost the same as those following STM measurements (=2.80 Å) with the difference close to 1%. Table 3 presents obtained changes in the Pt(111) geometry induced by presence of a two-dimensional gold layer. Namely, we find the top interlayer spacing $d_{.}^{Pt-Au}$ noticeably expanded by +2.16% with respect to the ideal platinum bulk value (=2.31 Å). The calculated value of the surface free energy of gold layer equals to γ_Au_ = 1.481 J/m^2^, and it is in very good agreement with the value of the surface free energy of Au(111) mentioned in literature (1.283 J/m^2^ < γ_Au(111)_ < 1.506 J/m^2^) [64,65,66,67,68].

**Table 2:** Calculation results of one Au atom adsorption on the Pt(111) surface. E_ads_—adsorption energy; *r_NN_* and *r_NNN_* describe the distance to the nearest (NN) and next-nearest (NNN) neighbors. D → B means that after calculations gold atom has moved from the bridge position D towards the most favorable hollow *fcc* position B.

(111)	Site	*E_ads_* (eV)	*r_NN_* (Å)	*r_NNN_* (Å)
Au on top	A	+0.580	2.58	3.76
Hollow *fcc*	B	−0.578	2.74	3.89
Hollow *hcp*	C	−0.518	2.75	3.92
Bridge	D → B	−0.578	2.74	3.89

**Table 3:** Calculated distances ($d_{i-j}^{X-Y}$) between the atomic planes of the relaxed Au-Pt(111) system, and their percentage changes ($\Delta d_{i-j}^{X-Y}$) with respect to the ideal Pt bulk value (d_0_), for the slab with eight atomic layers (see slab and top view of the considered structure in Figure 6).

$d_{i-j}^{X-Y}$ (Å)	Adsorption Site B	
	8 Layers $\Delta d_{i-j}^{X-Y}$ (%)	
$d_{.}^{Pt-Au}$	2.36	+2.16
$d_{1-2}^{Pt-Pt}$	2.34	+1.30
$d_{2-3}^{Pt-Pt}$	2.31	-
$d_{3-4}^{Pt-Pt}$	2.32	+0.43
$d_{4-5}^{Pt-Pt}$	2.31	-
$d_{5-6}^{Pt-Pt}$	2.30	−0.43
$d_{6-7}^{Pt-Pt}$	2.33	+0.86
d_0_	2.31	-

#### 4.2.3. Density of States

**Figure 7:**
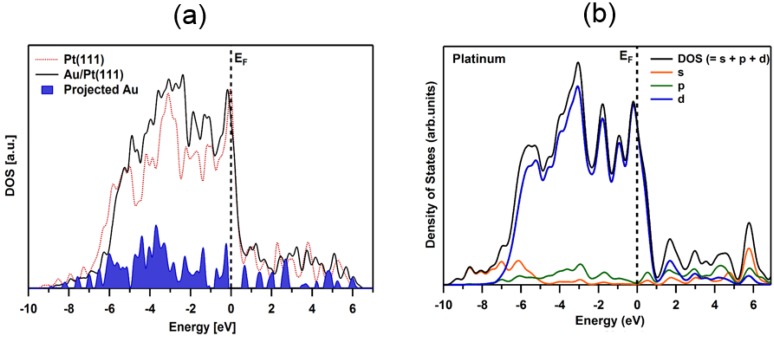
Density of states curves for gold on Pt(111): (**a**) Clean Pt(111) (red dotted line); 1 ML of Au on Pt(111) (black line). The projection of the adsorbed gold density of states is shaded in blue. (**b**) Clean bulk platinum (black line) and its components associated with s (orange line), p (green line) and d (blue line) orbitals. E_F_ denotes Fermi level.

The calculated electronic structure (density of states—DOS) for the studied adsorption system is presented in Figure 7. The DOS curve for bare Pt(111) and Pt(111) covered by Au is displayed in Figure 7a as red dotted and black line, respectively. In case of Au/Pt(111) surface, the DOS curve was obtained by considering gold atoms sitting in the most stable hollow *fcc* positions. Density of states distributions of Pt(111) and Au/Pt(111) systems were calculated for seven (clean platinum) and eight (one gold monolayer on platinum) atomic layers, respectively. In the case of density of states for clean Pt(111) surface, our results are in very good agreement with previous theoretical studies [73,74,75,76,77]. Changes in the electronic properties of our Au/Pt(111) system, compared to Pt(111), are visible. In particular, noticeable increase in the intensity of occupied states in the energy range between −5 and −1 eV, and slight change of the DOS shape after including of one gold layer into calculations. Both alterations, mainly attributed to the interaction of Au atoms with Pt(111) surface [39,40], are represented by the projection of the adsorbed gold density of states in Figure 7a. Density of states distribution calculated for the bulk platinum presented in Figure 7b, confirms well that the electronic structure of platinum is dominated by d state within the whole considered energy range.

## 5. Conclusions

In this work, experimental and theoretical studies of the geometrical and electronic properties of (111) surface of the ordered Au-Pt adsorption system have been presented. The analysis of LEED and STM measurements indicates that for a coverage below 1 ML, two-dimensional growth of the first Au monolayer takes place. Based on LEED results, no change of the lattice constant after gold adsorption was observed. The topography of the obtained STM images of Pt(111) and Au/Pt(111) surfaces on the level of the atomic resolution demonstrate that the surface structures have hexagonal arrangement of atoms and that the surface lattice constant is equal to the distance between the nearest platinum surface atoms (=2.80 Å). This is in very good agreement (close to 1%) with our presented DFT calculations, where the distances between the nearest atoms in the case of bare Pt(111) and Au/Pt(111) surface equal to 2.83 Å. It was shown that the first and second interlayer spacings of the clean Pt(111) surface were determined to be expanded by +0.87% and contracted by −0.43%, respectively. The calculated adsorption energy of the Au atom on the Pt(111) surface is dependent on the adsorption site, and there is a preference for a hollow *fcc* site (E_ads_ = −0.578 eV). In the presence of gold layer on the Pt(111) surface, the top interlayer spacing was found expanded by +2.16% with respect to the ideal bulk value. Density of states for the Pt(111) surface present very good agreement with previous literature studies, while observed changes in the electronic properties of the Au/Pt(111) system below the Fermi level are mainly connected to the interaction of Au atoms with Pt(111) surface.
